# Residential Greenness and Birthweight in the State of Massachusetts, USA

**DOI:** 10.3390/ijerph15061248

**Published:** 2018-06-12

**Authors:** Kelvin C. Fong, Itai Kloog, Brent A. Coull, Petros Koutrakis, Francine Laden, Joel D. Schwartz, Peter James

**Affiliations:** 1 Department of Environmental Health, Harvard T. H. Chan School of Public Health, Boston, MA 02115, USA; petros@hsph.harvard.edu (P.K.); francine.laden@channing.harvard.edu (F.L.); jschwrtz@hsph.harvard.edu (J.D.S.); 2 Department of Geography and Environmental Development, Ben-Gurion University of the Negev, Beer Sheva 653, Israel; ikloog@bgu.ac.il; 3 Department of Biostatistics, Harvard T. H. Chan School of Public Health, Boston, MA 02115, USA; bcoull@hsph.harvard.edu; 4 Department of Epidemiology, Harvard T. H. Chan School of Public Health, Boston, MA 02115, USA; 5 Channing Division of Network Medicine, Brigham and Women’s Hospital and Harvard Medical School, Boston, MA 02115, USA; 6 Department of Population Medicine, Harvard Medical School and Harvard Pilgrim Health Care Institute, Boston, MA 02115, USA; pjames@hsph.harvard.edu

**Keywords:** natural environment, greenness, health inequities, birth outcomes, birthweight, prenatal exposure

## Abstract

Natural vegetation, or greenness, may benefit maternal health and consequently, fetal growth, by providing opportunities for physical activity and psychological restoration, and decreasing detrimental environmental exposures. We retrieved Massachusetts Birth Registry data from 2001–2013 and investigated the association between residential greenness and birthweight in full-term births (≥37 weeks gestation). We calculated average residential greenness during pregnancy using 250 m normalized difference vegetation index (NDVI) from satellites. We estimated associations between greenness and continuous birthweight, term low birthweight (TLBW: <2500 g), and small for gestational age (SGA: <10th percentile of birthweight stratified by sex and gestational age) adjusted for individual and neighborhood covariates and considered nonlinearity and effect modification. Higher greenness exposure was associated with higher birthweight with stronger associations in the lower than higher range of greenness. Greenness was associated with lower odds of TLBW (OR 0.98; 95% CI 0.97, 0.99 per 0.1 increase in NDVI) and SGA (OR 0.98; 95% 0.97, 0.99) and associations varied by population density (TLBW) and socioeconomic status (TLBW, SGA). Our results suggest that greenness is beneficial to fetal growth exhibited by higher birthweight and lower odds of TLBW and SGA. Unlike prior studies, associations with TLBW and SGA appeared stronger among those with higher socioeconomic status.

## 1. Introduction

Lower birthweights are associated with higher risks for cardiovascular and respiratory disease, diabetes, obesity, and premature mortality [1,2,3,4,5]. Recently, there has been growing interest in how maternal exposure to nature may affect birthweight [6,7,8,9,10,11,12,13]. Prior studies found that increased maternal exposure to residential greenness, or natural vegetation around the home, was positively associated with birthweight. Greenness around the maternal residence is thought to improve health through mitigating exposure to air pollution, noise, and extreme temperatures; restoring mental health; and promoting physical activity and social engagement [14]. Vegetation may buffer and reduce exposure to air pollution, noise, and heat [15,16,17,18]. Contact with greenness may also relieve stress and mental fatigue [19,20]. Higher residential greenness may provide increased access to recreational space and is associated with healthy behaviors such as physical activity and social contact [19,21,22,23]. Through these pathways, higher residential greenness may improve maternal health during pregnancy, which, as a consequence, may lead to a healthy fetal environment.

There remain gaps in understanding the relationship between residential greenness and birthweight. Although most prior studies showed positive associations between greenness and higher birthweights [24], a few found null or negative associations [6,9]. The results from one recent study showed a positive association in Portland, Oregon, but a negative association in Austin, Texas, suggesting that there may be regional variation in how greenness is related to birthweights, perhaps due to differences in vegetation species type [9]. Thus, it is important to investigate these associations in additional study settings. Furthermore, prior studies found that associations may differ depending on the maternal socioeconomic status (SES) or the SES of the neighborhood of maternal residence [24]. Researchers have also found different associations depending on the population density near the residence [7,25]. We therefore, investigated the relationship between residential greenness and birthweight for all births in Massachusetts from 2001 to 2013. To further the understanding of this relationship, we assessed possible nonlinear associations by using natural splines. Moreover, as individual and neighborhood SES, as well as population density, may impact how mothers interact with residential greenness, we considered effect modification by SES indicators such as maternal education, government support for prenatal care, and Census median household income, as well as population density [24,26].

## 2. Materials and Methods

### 2.1. Data

#### 2.1.1. Study Population

We used data from the Massachusetts Birth Registry from 1 January 2001 to 31 December 2013 (*n* = 978,225). We excluded records that had missing residence information (*n* = 23,943), those that were not live births (*n* = 8621), not singletons (*n* = 42,186), not full-term (<37 weeks; *n* = 77,036), and below 500 g in birthweight (*n* = 772). A further 45,232 were excluded due to missing covariate data, leading to a final sample size of 780,435. Similar exclusion criteria were applied in recent studies [6,7,8]. Births with maternal residence address were geocoded by the Massachusetts Department of Public Health against TomTom Multinet using AccuMail (Smartsoft, Westlake Village, CA, USA) address and zip code. The use of birth data was approved by the Massachusetts Department of Public Health and the human subjects committee at the Harvard T. H. Chan School of Public Health.

#### 2.1.2. Exposure: Residential Greenness

To assess residential greenness, we used remote sensing data from the Moderate Resolution Imaging Spectroradiometer (MODIS) aboard the Terra and Aqua satellites operated by the United States National Aeronautics and Space Administration. We derived the normalized difference vegetation index (NDVI) at 250 m resolution from these data [27]. Chlorophyll in plants absorbs visible light between 0.4 and 0.7 μm in wavelength for photosynthesis while reflecting near-infrared light between 0.7 and 1.1 μm in wavelength. NDVI calculates a ratio of difference between visible and near-infrared divided by the sum of visible and near-infrared light to estimate the quantity of vegetation in a given area. Higher NDVI values are indicative of more vegetation, and thus higher greenness. For each birth, we assigned an average NDVI value, referred to as greenness, representative of the maternal residential greenness exposure during pregnancy. First, using the geocode of each birth, we determined the 250 m by 250 m grid pixel in which the mother reported to have resided at the time of the birth. We chose 250 m as the spatial resolution since it captures the local residential greenness outside the mother’s home and is most accessible [8]. After the grid pixel of each birth was assigned, we determined, again for each birth, the exposure window as time during which each mother was pregnant using the clinical gestational age reported in the birth record. We then calculated the average of NDVI values occurring during the pregnancy for each birth from a time-varying NDVI dataset containing measurements from January, April, July, and October representing winter, spring, summer, and fall. This exposure assignment is similar to those used in recent studies [7,8].

#### 2.1.3. Outcomes

Continuous birthweight was measured in grams shortly after the time of birth. We also defined term low birthweight (TLBW) as birthweight less than 2500 g in a full-term birth, and small for gestational age (SGA) as birthweight below the 10th percentile of the birthweights for the newborn’s sex and gestational age. Gestational age was determined by a medical professional during a prenatal clinical examination before birth.

#### 2.1.4. Covariates

Model covariates were chosen a priori and selected for their potential to confound the relationship between exposure to greenness and birthweight. They were retrieved or ascertained from the birth records and include maternal age (years), maternal race (White, Black, Asian, American Indian, other), maternal marital status (married, not married), maternal smoking prior to or during pregnancy (yes, no), maternal highest level of education attained (less than high school, high school, some college, college, advanced degree beyond college), parity (first-born, not first-born), maternal diabetes (yes, no), gestational diabetes (yes, no), maternal chronic high blood pressure (yes, no), maternal high blood pressure during pregnancy (yes, no), Kessner index of adequacy of prenatal care (adequate, intermediate, inadequate, no prenatal care) [28], birth mode of delivery (vaginal, forceps, vacuum, first caesarian birth, repeat caesarian birth, vaginal birth after caesarian birth), clinical gestational age (weeks), year of birth (one of 2001 to 2013), newborn sex (male, female), government support for prenatal care (yes, no), and season of birth (spring: birth date between 21 March and 20 June, summer: 21 June to 20 September, autumn: 21 September to 20 December, winter: 21 December to 20 March). We also adjusted for 2006–2010 American Community Survey (ACS) estimates of black population proportion and median household income (10,000s of US dollars per year) at the Census block group level [29]. We included particulate air pollution under 2.5 µm in diameter (PM_2.5_), since maternal exposure to PM_2.5_ during pregnancy has been shown to be negatively associated with birthweight [11,30,31,32,33,34]. Each birth’s PM_2.5_ exposure was calculated by averaging daily PM_2.5_ predictions of the 1 km × 1 km grid pixel of the maternal residential address during the entire pregnancy. The PM_2.5_ data were predicted by a hybrid model that used satellite-derived aerosol optical depth and performed consistently with an out-of-sample R^2^ above 0.8 [35]. We included population density as a covariate defined as over versus under 1000 people per square mile at the Census tract reported in the 2006–2010 ACS estimates [29]. This threshold came from the US Census’s reference manual and has previously been found to be a modifier of associations between greenness and other health outcomes [25,36].

### 2.2. Analysis

We first assessed the relationship between greenness and continuous birthweight. We constructed a nonlinear model with a natural spline for NDVI and additionally adjusted for all covariates. To test whether the nonlinear model provided a better fit, we used the likelihood-ratio test and compared it to a simpler model with a linear term for NDVI instead. Next, we considered possible effect modification by adding interaction terms between NDVI and each of newborn sex, maternal education, government support for prenatal care, Census block group median household income, Census block group percent black population, and population density. We also ran analyses excluding PM_2.5_ as a covariate to assess whether findings were mediated by PM_2.5_ exposure. As TLBW and SGA are binary outcomes, we built logistic models estimating average differences in the odds of TLBW or SGA associated with higher levels of greenness. We followed a similar process in modeling TLBW and SGA, testing first for nonlinearity then for effect modification. We conducted our analyses using the statistical language R [37]. Statistical significance was set at the 0.05 alpha level with 2-sided tests.

## 3. Results

### 3.1. Summary Statistics

Table 1 shows descriptive statistics in the study population. For the births in our analysis, NDVI ranged from −0.14 to 0.93 with an interquartile range (IQR) of 0.22. The mean birthweight was 3441 g and the mean residential greenness exposure was 0.49 NDVI. About one-third of the mothers received government support for prenatal care, three-quarters reported living in high density population areas, and more than half received at least some college education. Birthweight, maternal age, Census block group median household income, being first-born, having a married mother, having a white mother, receiving adequate prenatal care, chronic and gestational diabetes, and having a mother with higher education were higher in those born to mothers with higher residential greenness exposure (Table 1). Conversely, Census block group black population proportion, receiving government support for prenatal care, and having a mother who had diabetes were lower with higher greenness.

### 3.2. Continuous Birthweight

**Figure 1:**
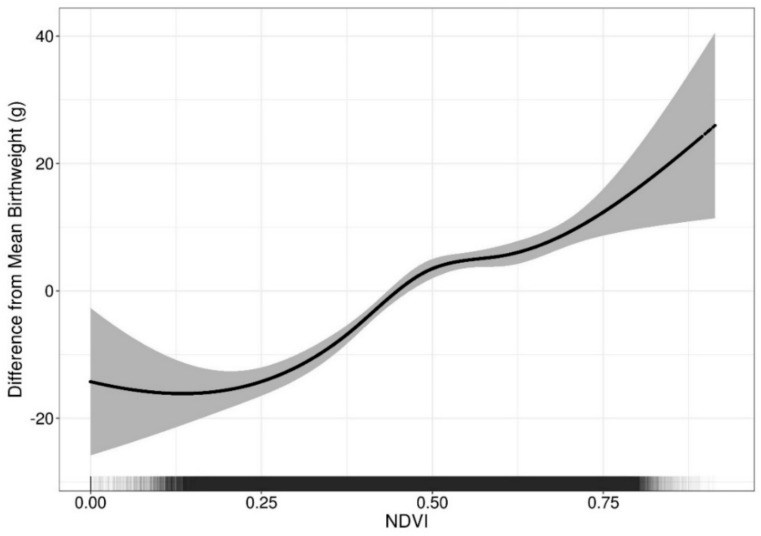
Nonlinearity in the Association between Greenness and Birthweight. Black line shows the predicted difference from mean birthweight (3441 g) in a range of normalized difference vegetative index (NDVI) values, given that all other covariates are at their respective means. 95% confidence intervals are shaded in gray. Rug plot on x-axis represents density of observed NDVI values. Nonlinear model with a natural spline for NDVI adjusted for the following covariates: particulate matter (PM_2.5_), maternal age, race, smoking prior to or during pregnancy, education, parity, chronic diabetes, gestational diabetes, chronic high blood pressure, gestational high blood pressure, Kessner index of adequacy of prenatal care, birth mode of delivery, clinical gestational age, newborn sex, government support for prenatal care, season of birth, year of birth, Census black population proportion, Census median household income, and population density.

Higher greenness was associated with higher birthweight after adjustment for all model covariates (Figure 1). A nonlinear model with a natural spline for NDVI was more appropriate for describing the association than the model with a linear term for NDVI (likelihood ratio test for a nonlinear association: *p* < 0.05). Figure 1 shows that between 0.25 and 0.50 NDVI, the estimated difference in birthweight per 0.1 NDVI increase was 6.69 (95% CI: 5.18, 8.19) g; between 0.50 and 0.75 NDVI, the estimated difference in birthweight per 0.1.

NDVI increase was 2.06 (95% CI: 0.09, 4.12) g. Estimated differences below 0.25 or above 0.75 NDVI were not statistically significant. Results were similar in a nonlinear model that excluded PM_2.5_ as a covariate, suggesting that findings were not substantially mediated by PM_2.5_ (Table A1). We did not find evidence to support effect modification by newborn sex, maternal education, government support for prenatal care, Census block group black population proportion, Census median household income, nor population density.

### 3.3. Term Low Birthweight (TLBW)

**Figure 2:**
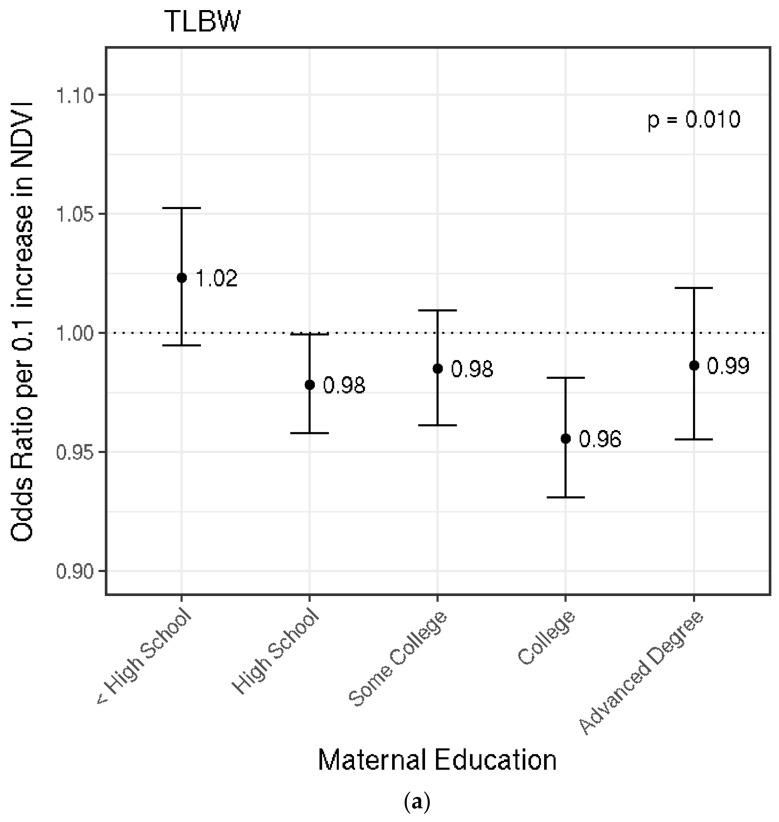

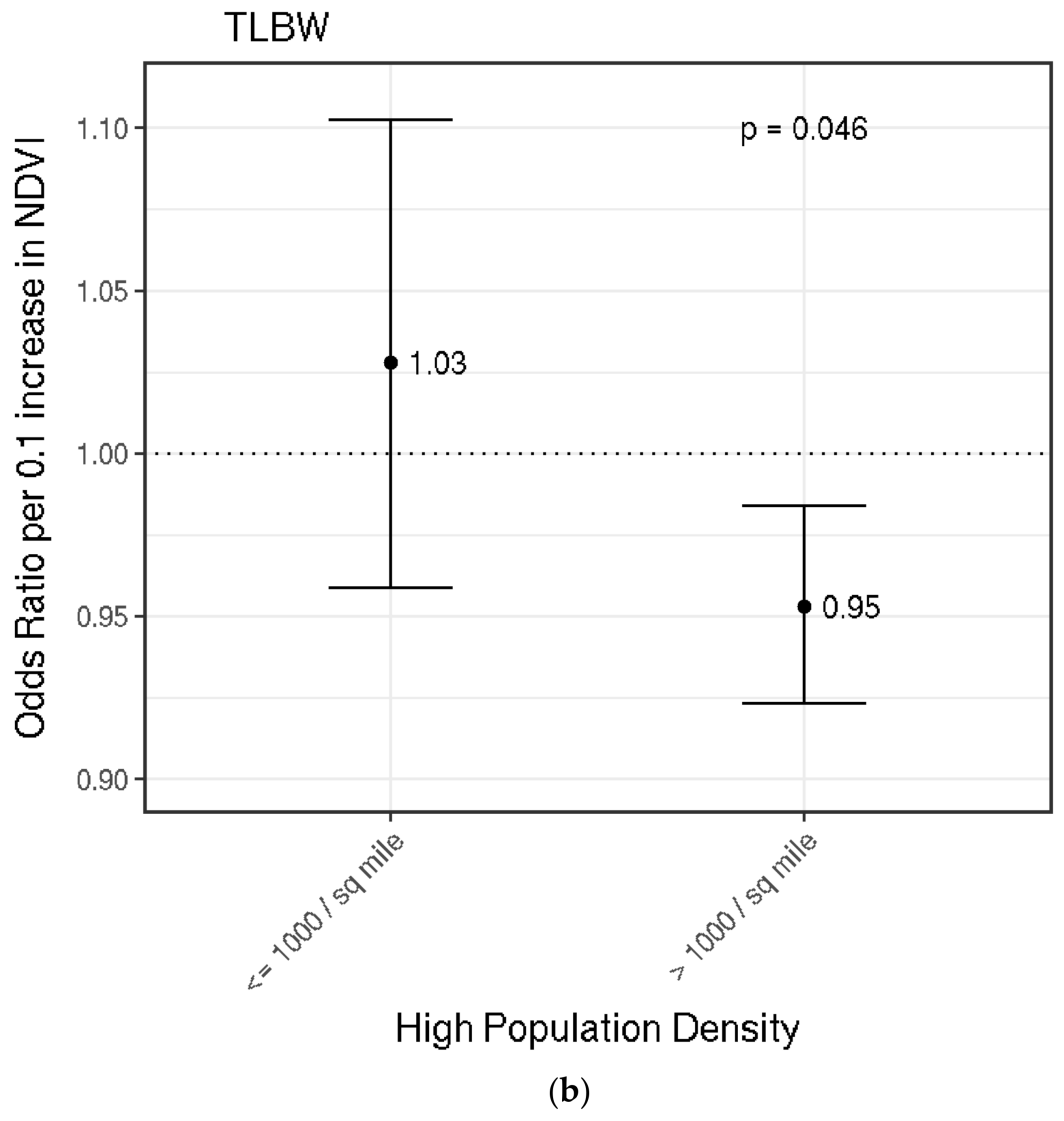
Effect Modification by Maternal Education in the Association between Greenness and Term Low Birthweight (TLBW). Odds ratios and 95% confidence intervals per 0.1 increase in NDVI are shown. Dotted line at 1.0 represents a null association. *p*-values for interaction between greenness and the effect modifier are shown near the top right of each plot. (**a**) shows the odds ratio estimates for TLBW stratified by maternal education; (**b**) shows the odds ratio estimates for TLBW stratified by population density. Each model adjusted for the following covariates unless it was the stratifying variable: particulate matter (PM_2.5_), maternal age, race, smoking prior to or during pregnancy, education, parity, chronic diabetes, gestational diabetes, chronic high blood pressure, gestational high blood pressure, Kessner index of adequacy of prenatal care, birth mode of delivery, clinical gestational age, newborn sex, government support for prenatal care, season of birth, year of birth, Census black population proportion, Census median household income, and population density.

Increased greenness was associated with lower odds of TLBW. On average, the odds ratio (OR) for TLBW per a 0.1 increase in NDVI was 0.98 (95% CI: 0.97, 0.99). A nonlinear logistic model with a natural spline for greenness did not provide a better fit over the model with a linear term for greenness. The OR for TLBW was similar in a model that excluded PM_2.5_ as a covariate, suggesting that findings were not mediated by PM_2.5_ (Table A1). We found evidence to support effect modification by maternal education and population density (*p* < 0.05). Estimated ORs stratified by maternal education per 0.1 increase in NDVI are shown in Figure 2a, demonstrating that greenness was associated with lower odds of TLBW for higher levels of maternal education. For instance, the estimated OR among those born to college-educated mothers was 0.96 (95% CI: 0.93, 0.98), while the OR for those born to mothers with less than a high school education was 1.02 (95% CI: 0.99, 1.05). Effect modification by population density was statistically significant (*p* = 0.046) and the estimated association among those born to mothers who lived in high population density areas was stronger (OR 0.95; 95% CI: 0.92, 0.98).

### 3.4. Small for Gestational Age (SGA)

**Figure 3:**
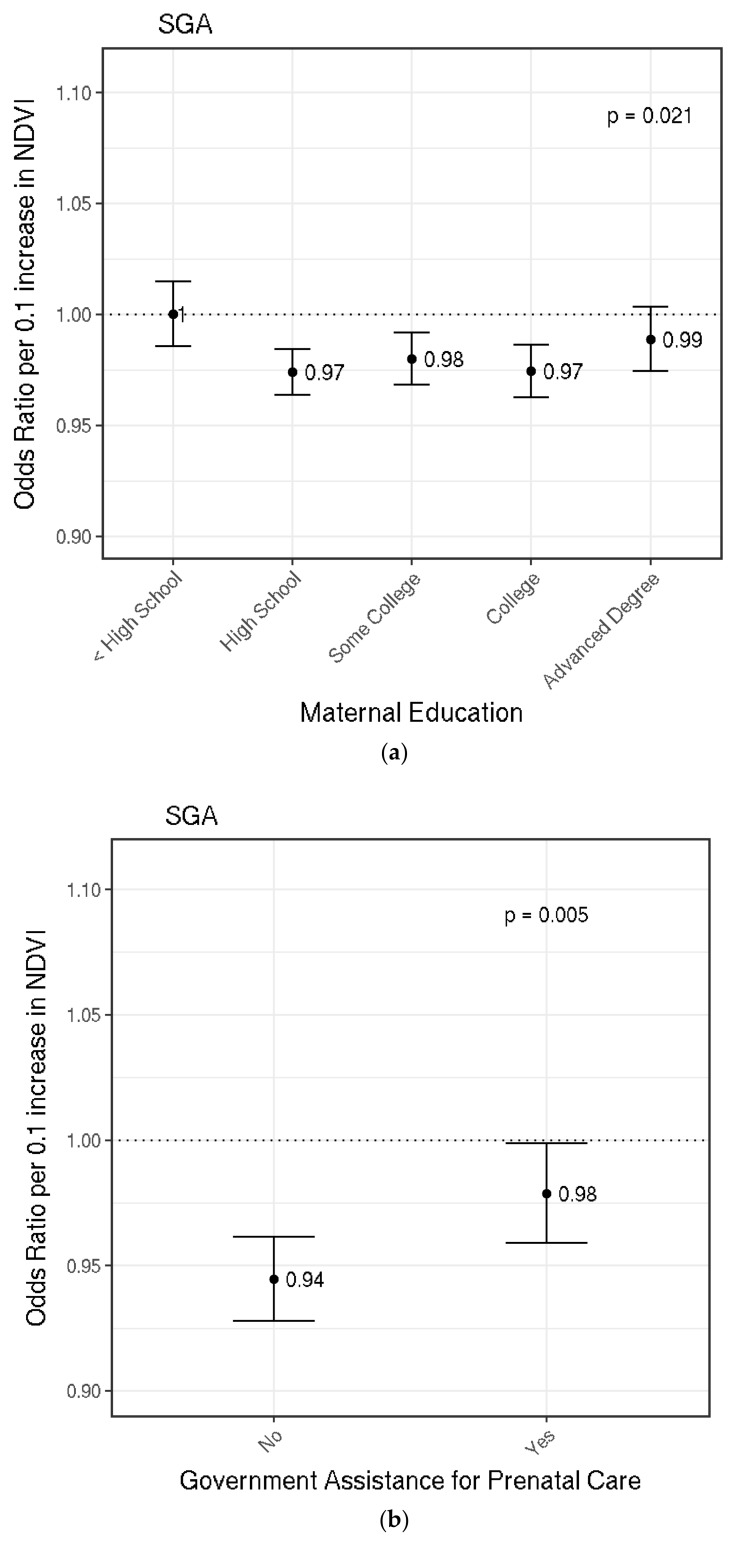

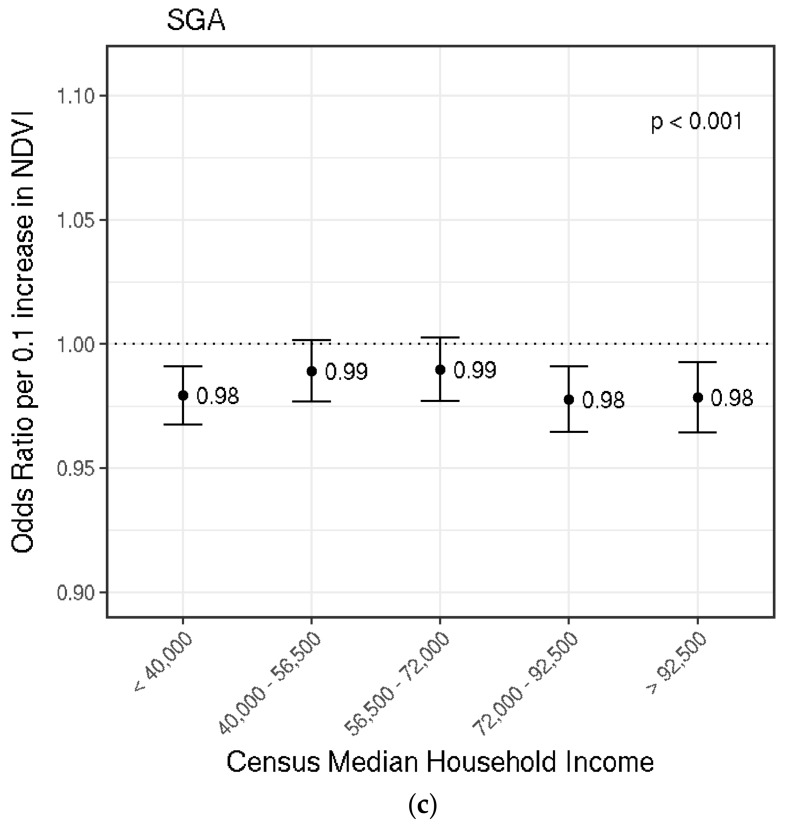
Effect Modification by Maternal Education in the Association between Greenness and Small for Gestational Age (SGA). Odds ratios and 95% confidence intervals per 0.1 increase in NDVI are shown. Dotted line at 1.0 represents a null association. *p*-values for interaction between greenness and the effect modifier are shown near the top right of each plot. (**a**) shows the odds ratio estimates for SGA stratified by maternal education; (**b**) shows the odds ratio estimates for SGA stratified by government assistance for prenatal care; (**c**) shows the odds ratio estimates for SGA stratified by Census block group median household income. Each model adjusted for the following covariates unless it was the stratifying variable: particulate matter (PM_2.5_), maternal age, race, smoking prior to or during pregnancy, education, parity, chronic diabetes, gestational diabetes, chronic high blood pressure, gestational high blood pressure, Kessner index of adequacy of prenatal care, birth mode of delivery, clinical gestational age, newborn sex, government support for prenatal care, season of birth, year of birth, Census black population proportion, Census median household income, and population density.

Increased greenness was associated with lower odds of SGA. On average, the OR for SGA per a 0.1 increase in NDVI was 0.98 (95% CI: 0.97, 0.99) in fully adjusted models. A nonlinear logistic model with a natural spline for greenness did not provide a better fit than a linear term for greenness. The OR for SGA was similar in a model that excluded PM_2.5_ as a covariate, suggesting that findings were not mediated by PM_2.5_ (Table A1). We observed effect modification by maternal education, government support for prenatal care, and Census block group median household income (*p* < 0.05). Estimated ORs stratified by maternal education are graphed in Figure 3a, stratified by government support for prenatal care in Figure 3b, and stratified by Census block group median annual household income in Figure 3c. Similar to findings for TLBW, ORs for SGA for those born to mothers with higher education attainment were stronger than those with mothers with less than a high school education. In a separate model that estimated the odds of SGA stratified by government assistance for prenatal care, the estimated association was stronger among those without government assistance for prenatal care (OR 0.94; 95% CI: 0.93, 0.96) than those with assistance (OR 0.98; 95% CI: 0.96, 1.00). Finally, associations between greenness and SGA were generally strongest among those whose mothers lived in Census block groups with the highest median household income, although associations did not monotonically increase with increasing median household income. For SGA, we did not observe effect modification by population density.

## 4. Discussion

In our analysis of Massachusetts singleton full-term live births from 2001 to 2013, we found a positive nonlinear association between maternal exposure to residential greenness during pregnancy and continuous birthweight. We observed stronger associations in the lower range of greenness (0.25–0.50 NDVI) than the higher range of greenness (0.50–0.75 NDVI) (Figure 1). We observed linear inverse associations between greenness and the odds of TLBW or SGA. The association between greenness and TLBW or SGA was modified by SES indicators. For TLBW, the strongest associations were observed among those born to mothers with higher educational attainment. Associations did not change substantially when PM_2.5_ was excluded from analyses, suggesting that the relationship between greenness and birthweight outcomes were not mediated by PM_2.5_. Maternal education, government assistance for prenatal care, and Census block group median annual household income modified the relationship between greenness and SGA, with the strongest findings generally among mothers with higher SES. Stratified analyses also demonstrated somewhat stronger associations between greenness and TLBW among those living in high population density areas.

Several mechanisms could explain the association between greenness and higher birthweights. The main pathways are mitigating harmful environmental exposures, restoring mental capacity, and providing a setting for social interactions as well as physical activity [14,19,26]. Vegetation such as trees may filter the air and serve as physical barriers to heat, air pollution, and noise [24,26]. Traffic air pollution has been shown to be considerably lower in areas of high greenness [38]. Multiple studies have found that cognitive function and mental capacity improve with increased greenness exposure [14,19]. Moreover, since greenness often occurs in recreational spaces such as parks, high greenness is often indicative of proximity to places where people can meet socially or exercise, improving health through social cohesion and physical activity. Finally, although research directly looking at physiological responses to greenness is scarce, experiments have shown that proximity to greenness is associated with greater autonomic activity via measurements of heart rate variability [39]. Other physiological findings include less pronounced response to stress and better immune function [40]. Although the exact mechanism remains unclear, living in areas of higher greenness during pregnancy can result in unperturbed fetal growth through improved fetal oxygenation [41]. Fetal hypoxia is a known cause of intrauterine growth restriction and mothers and increased greenness can potentially decrease this by improving overall maternal health through afore-mentioned mechanisms such as increased physical activity or decreased exposure to environmental stressors such as heat and noise [14,41]. In summary, conditions for a healthy fetal environment such as sufficient fetal perfusion and oxygenation are more likely to be met in mothers living in areas of higher greenness [41], ultimately resulting in unperturbed fetal growth and increased birthweight [10,14,24].

The majority of previous studies also found a positive association between greenness and continuous birthweight [24], although our results fall in the lower range of existing estimates. Large positive associations were found in British Columbia, where a 0.1 NDVI increase was associated with a 20.6 (95% CI: 16.5, 24.7) g increase in birthweight [13]; in a study in Tel Aviv, Israel, where a 0.06 NDVI increase was associated with a 19.2 (95% CI: 13.3, 25.1) g increase in birthweight [42]; and in a pooled analysis of four Spanish birth cohorts a 38.3 (95% CI: 17.1, 59.5) g per 0.19 NDVI increase [10]. It is possible that due to our conservative adjustment for different covariates, such as population density and air pollution, our estimates may be different from other analyses; however, we found that the associations between greenness and birthweight outcomes were similar when omitting PM_2.5_ as a covariate (Table A1). We found a positive nonlinear relationship between greenness and continuous birthweight (Figure 1). In the lower range of greenness between 0.25 and 0.50 NDVI, the association was strongly positive. In the higher range of greenness between 0.50 and 0.75 NDVI, the association was positive but about half in magnitude. The characterization of a nonlinear dose-response relationship between greenness and continuous birthweight has not been previously reported unlike nonlinearity for other outcomes such as physical activity [43]. In some prior studies of greenness and birthweight, it was not possible to determine the shape of the dose-response relationship since greenness exposure was classified into quantiles of NDVI [8,24]. Of some studies that considered NDVI on the continuous scale, one study used quadratic and cubic splines in their analysis, but did not find a departure from linearity [7]. It is possible that the pathways through which greenness impacts birthweight in the lower range of greenness are different from those in the higher range of greenness. This may explain why positive, null, or even negative associations were found in existing literature [24]. A recent study of over three million birth records in Texas found only a 1.9 (95% CI: 0.1, 3.7) g increase in birthweight when comparing those in the highest quartile of greenness (NDVI > 0.52) versus those in the lowest quartile (NDVI < 0.37) [8]. Another study by Cusack et al. found a positive association for those born in Portland, Oregon, but a negative association for those born in Austin, Texas [9]. Some studies did not find statistically significant changes in birthweight with increased greenness or proximity to green spaces [6,44].

Importantly, nonlinearity between greenness and health has policy implications. When considering potential benefits to birthweight by increasing greenness, it may be more beneficial to focus on developing climate-appropriate areas from low greenness to medium greenness, as opposed to developing from medium greenness to high greenness. However, as this is a novel finding, further investigation of the dose-response relationship between greenness and birthweight is needed.

Overall, higher greenness was associated with lower odds of TLBW and SGA. This was expected since there was a positive association between greenness and continuous birthweight. The association between increased greenness and lower odds of TLBW and SGA has previously been reported [7,12,13,24,42]. With TLBW and SGA, we found the association with greenness to be linear and modified by population density (TLBW), maternal highest education attainment (TLBW, SGA), government support for prenatal care (SGA), and Census block group median household income (SGA). For population density, we found associations among those who reported living in high population density areas but not among those who reported living in low population density areas. A similar finding was published by Casey et al., who reported strong associations in cities but not in boroughs and townships (lower population density than cities) [7]. In a separate analysis, the association between greenness and depressive symptoms in adolescents was also reported to be stronger among those in high population density areas but not in low population density areas [25]. The mechanisms behind why population density modifies associations with greenness are unclear, but it was possible that greenness helped decrease detriments from exposures that were present in high population density areas but not in low population density areas [24,26]. Those with higher education attainment or without government support for prenatal care had higher individual SES [45,46]; higher Census block group median household income was also indicative of higher neighborhood SES. Our results stratified by SES did not demonstrate a monotonic relationship; however, they were suggestive of stronger associations among those born to mothers of higher individual or neighborhood SES. This finding contrasts those previously reported in the literature [14,24], which concluded that greenness associations were stronger among those with lower SES. Individual and neighborhood SES can alter how one interacts with greenness, such as accessing nearby areas of greenness. It was possible that lower individual or neighborhood SES hinders true access to areas of greenness, thus leading to weaker associations [14]. Those with lower individual SES might not be as likely to access areas of surrounding greenness or there might be safety concerns in areas of lower neighborhood SES discouraging access of green areas. In summary, the overall associations between greenness and lower odds of TLBW and SGA was consistent with previous studies. However, patterns in how SES indicators modified these associations contrast most previous findings, demonstrating that how SES impacts potential health effects of greenness is variable, complex, and warrants further investigation.

Our study had strengths and limitations. With a final sample size of 780,435, which represents one of the largest studies on the relationship between greenness and birthweight to date [8,24], the study had high statistical power and the ability to assess nonlinear associations as well as effect modification by SES indicators. For greenness exposure assignment, we used multiple temporally-matched measurements to determine exposure during pregnancy. Therefore, we captured the time-varying nature of greenness which is likely more accurate than prior studies that used only a single satellite image from, for instance, one summer day [6,12,44]. This likely reduced bias from exposure misclassification. That said, exposure assignment was limited by basing all calculations on maternal residence reported at the time of birth, and mothers may have changed addresses since conception. We could not check if there were errors in maternal residence or other included model covariates, but we expect the bias from such measurement error to be non-differential. Furthermore, some births in the complete database had to be excluded because of missing maternal residence information. But since there was relatively low exclusion due to missing maternal residence, bias from this source was likely minimal. Another limitation with greenness exposure was our choice to use NDVI. Although it is an established measure of greenness [27], it does not provide information on the specific types of vegetation such as trees, grass, or shrubs, nor does it describe accessibility of green spaces. How mothers interacted with greenness depending on the type of vegetation could be very different and without this information, we were unable to devise specific recommendations on types of vegetation that might be most associated with higher birthweights and lower odds of TLBW or SGA [14,24,26]. In fact, the variety of estimated associations for greenness in the existing literature could be due to differences in vegetation species types across study settings. By using 250 m as the spatial resolution, we assumed that pathways through which greenness influences fetal growth were limited the mothers’ activity in the immediate area around their residence. While prior studies found no considerable differences in associations when using 100, 250, 500 and 1250 m spatial resolutions [6,7,42], it is unclear that only local residential greenness influenced fetal growth. Finally, the generalizability of some of our findings was limited due to the demographics of the study population. Almost three-quarters of the mothers were white and over three-quarters reported living in high population density areas. Our finding of stronger associations among those with higher SES, which was inconsistent with most existing literature on greenness and birth outcomes [24], could be explained by differences in demographics between study populations.

## 5. Conclusions

Our findings from a birth registry in Massachusetts from 2001–2013 support that maternal exposure to residential greenness may benefit fetal growth, associated with higher birthweights and lower odds for TLBW or SGA. The association between greenness and continuous birthweight was nonlinear with stronger associations in the lower range of greenness compared to the higher range. Greenness was linearly associated with lower odds of TLBW or SGA and these associations were modified by indicators of SES. The overall pattern in associations suggest stronger associations among those born to mothers with higher individual SES or living in areas of higher SES, a finding that contrasts conclusions from other studies. A possible explanation is that greenness may have variable effects depending on study settings and demographics of the study population. To build upon our understanding of how greenness impacts human health, future work on birth outcomes should further explore nonlinearity and effect modification by SES.

## Figures and Tables

**Table 1:** Characteristics of Massachusetts full-term, singleton live births from 2001 to 2013 (*n* = 780,435).

Variable	Overall	NDVI Q1 (Mean < 0.38)	NDVI Q4 (Mean > 0.60)
*Continuous Variables (mean ± s.d.)*			
Residential Greenness (NDVI)	0.49 ± 0.15	0.29 ± 0.07	0.67 ± 0.05
Birthweight (g)	3441 ± 472	3400 ± 472	3477 ± 468
PM_2.5_ (µg/m^3^)	10.1 ± 1.4	10.5 ± 1.4	9.8 ± 1.3
Clinical Gestational Age (weeks)	39.3 ± 1.2	39.4 ± 1.2	39.3 ± 1.1
Mother’s Age (years)	30.1 ± 6.0	28.8 ± 6.2	31.3 ± 5.7
Census Black Population Proportion	0.08 ± 0.15	0.11 ± 0.17	0.04 ± 0.10
Census Median Household Income ($10,000s)	6.8 ± 3.3	5.2 ± 2.7	8.2 ± 3.3
*Binary and Categorical Variables (%)*			
Newborn Female Sex	49.0	49.0	49.1
Term Low Birthweight	2.1	2.1	1.8
Small for Gestational Age	9.1	9.1	8.3
Parity: First-born	54.7	54.7	57.8
Mother Married	68.9	68.9	78.7
Government Support for Prenatal Care	33.0	33.0	21.5
Maternal Smoking	13.7	13.7	13.2
Gestational Diabetes	4.0	4.0	3.8
Other Diabetes	0.9	0.9	0.8
High Blood Pressure during Pregnancy	3.3	3.3	3.3
Chronic High Blood Pressure	1.2	1.2	1.1
Census Tract Population Density > 1000 people/sq. mile	76.8	94.9	53.0
Season of Birth			
Winter	24.4	24.4	28.2
Spring	25.1	25.1	14.1
Summer	26.0	26.0	16.1
Fall	24.5	24.5	41.7
Mode of Delivery			
Vaginal	65.5	65.5	64.4
Forceps	0.6	0.6	0.6
Vacuum	3.6	3.6	3.5
First Caesarian Birth	16.7	16.7	16.4
Repeat Caesarian	12.1	12.1	13.5
Vaginal Birth after previous Caesarean Birth	1.6	1.6	1.7
Maternal Race			
White	71.9	71.9	85.4
Black	8.4	8.4	4.5
Asian	7.6	7.6	5.8
American Indian	0.2	0.2	0.3
Other	11.9	11.9	4.0
Kessner Index for Prenatal Care			
Adequate	78.6	78.6	81.9
Intermediate	17.0	17.0	14.6
Inadequate	3.3	3.3	2.5
No Prenatal Care	1.2	1.2	1.0
Maternal Education			
Less than High School	10.7	10.7	5.4
High School	24.0	24.0	19.0
Some College	22.3	22.3	23.1
College	26.3	26.3	32.6
Advanced Degree	16.7	16.7	20.0

## Appendix A

**Table A1:** Associations between Greenness and Continuous Birthweight, Term Low Birthweight, and Small for Gestational Age in Models omitting Particulate Matter (PM_2.5_) as a Covariate. Each model adjusted for the following covariates: maternal age, race, smoking prior to or during pregnancy, education, parity, chronic diabetes, gestational diabetes, chronic high blood pressure, gestational high blood pressure, Kessner index of adequacy of prenatal care, birth mode of delivery, clinical gestational age, newborn sex, government support for prenatal care, season of birth, year of birth, Census black population proportion, Census median household income, and population density.

	Association with 0.1 Increase in NDVI in Model Excluding PM_2.5_
**Continuous Birthweight**	
0.25–0.50 NDVI	6.92 (95% CI: 5.42, 8.42)
0.50–0.75 NDVI	2.28 (95% CI: 0.18, 4.38)
**Term Low Birthweight (TLBW)**	0.98 (95% CI: 0.97, 1.00)
**Small for Gestational Age (SGA)**	0.98 (95% CI: 0.98, 0.99)
